# A New Vista of Aldehyde Dehydrogenase 1A3 (ALDH1A3): New Specific Inhibitors and Activity-Based Probes Targeting ALDH1A3 Dependent Pathways in Glioblastoma, Mesothelioma and Other Cancers

**DOI:** 10.3390/cancers16132397

**Published:** 2024-06-28

**Authors:** Lorenzo Magrassi, Giulia Pinton, Sabino Luzzi, Sergio Comincini, Andrea Scravaglieri, Valentina Gigliotti, Bianca Laura Bernardoni, Ilaria D’Agostino, Francesca Juretich, Concettina La Motta, Silvia Garavaglia

**Affiliations:** 1 Neurosurgery, Dipartimento di Scienze Clinico-Chirurgiche e Pediatriche, Università degli Studi di Pavia, Fondazione IRCCS Policlinico S. Matteo, 27100 Pavia, Italy; sabino.luzzi@unipv.it (S.L.); andrea.scravaglieri01@universitadipavia.it (A.S.); 2 Istituto di Genetica Molecolare—CNR, 27100 Pavia, Italy; 3 Department of Scienze del Farmaco, University of Piemonte Orientale, Via Bovio 6, 28100 Novara, Italy; giulia.pinton@uniupo.it (G.P.); valentina.gigliotti@uniupo.it (V.G.); silvia.garavaglia@uniupo.it (S.G.); 4 Dipartimento di Biologia e Biotecnologie, Università di Pavia, 27100 Pavia, Italy; sergio.comincini@unipv.it; 5 Department of Pharmacy, University of Pisa, Via Bonanno 6, 56126 Pisa, Italy; bianca.bernardoni@phd.unipi.it (B.L.B.); ilaria.dagostino@unipi.it (I.D.); concettina.lamotta@unipi.it (C.L.M.)

**Keywords:** aldehyde dehydrogenase, glioblastoma, mesothelioma, cancers, ALDH1A3, retinoic acid, fluorescence-guided surgery

## Abstract

**Simple Summary:**

Aldehyde dehydrogenases of the subfamily 1A (ALDH1A) are enzymes involved in the synthesis of retinoic acid, which is necessary for the normal development and maintenance of epithelia, reproduction, memory, and immune function in adults. ALDH1A3, one of the enzymes that belong to the ALD1A subfamily, is also expressed at high levels in many human cancers like glioblastoma and mesothelioma. Herein, we review the role of ALDH1A3 in cancer, showing its relation with excessive proliferation, chemoresistance, and invasiveness. We also illustrate the current attempts to develop ALDH1A3-selective inhibitors and specific fluorescent probes that are potentially useful for cancer therapy and fluorescence-guided tumor resection.

**Abstract:**

Aldehyde dehydrogenases of the subfamily 1A (ALDH1A) are enzymes necessary for the oxidation of all-*trans* or 9-*cis* retinal to retinoic acid (RA). Retinoic acid and its derivatives are important for normal development and maintenance of epithelia, reproduction, memory, and immune function in adults. Moreover, in recent years, it has been demonstrated that ALDH1A members are also expressed and functional in several human cancers where their role is not limited to the synthesis of RA. Here, we review the current knowledge about ALDH1A3, one of the 1A isoforms, in cancers with an emphasis on two of the deadliest tumors that affect humans: glioblastoma multiforme and mesothelioma. In both tumors, ALDH1A3 is considered a negative prognostic factor, and its level correlates with excessive proliferation, chemoresistance, and invasiveness. We also review the recent attempts to develop both ALDH1A3-selective inhibitors for cancer therapy and ALDH1A3-specific fluorescent substrates for fluorescence-guided tumor resection.

## 1. Introduction

The multigene family of human aldehyde dehydrogenases (ALDHs) has 19 functional members that encode NAD(P)^+^-dependent enzymes devoted to the biotransformation and detoxification of multiple endogenous and exogenous aldehyde substrates [1,2]. Moreover, in the course of evolutionary history, several members of the ALDH superfamily have also acquired other enzymatic functions like esterase activity with a significant role in the bioactivation of nitrates but also non-enzymatic structural functions like acting as lens and corneal crystallins capable of quenching the effects of UV radiation [3].

Our review article is focused on the role in oncology of one important member of the ALDH1A subfamily, namely ALDH1A3. We also consider new diagnostic and therapeutic approaches based on the development of selective inhibitors and probes specific for ALDH1A3 [2,4].

The human ALDH1A subfamily encompasses three genes: *ALDH1A1* located on chromosome 9q21.13, *ALDH1A2* located on chromosome 15q21.3, and *ALDH1A3* located on chromosome 15q26.3. Phylogenetic analysis of the evolution of vertebrate ALDH1A genes suggests that *ALDH1A1* and *ALDH1A2* form a clade sister to *ALDH1A3* [5], confirming the specificity of *ALDH1A3*. *ALDH1A1*, *ALDH1A2*, and *ALDH1A3* have the lowest ratio of observed and expected variant numbers in the genome aggregation database (gnomAD) of all human ALDH genes. This suggests that, at least in humans, the genes of the ALDH1A subfamily have the lowest tolerance for loss-of-function mutations as compared to the gene members of the other ALDH subfamilies [1].

In mice, deletion of the *Aldh1a1* gene is viable and fertile, and *Aldh1a1*-deleted mice are protected against diet-induced obesity and insulin resistance [6]. On the contrary, genetic deletion of *Aldh1a2* [7] is embryonically lethal, and *Aldh1a3*-deleted mice die at birth due to choanal atresia which is responsible for severe respiratory distress and inability to thrive leading to the death of the animals in the immediate post-partum period [8]. Most alterations in *Aldh1a* knockouts have been traced to a complete or relative lack of retinoic acid (RA) at the necessary time points and tissues during development [8,9].

## 2. Structure and Catalysis of ALDHs

Besides various functions and natural substrates, ALDH enzymes also share a significant amino acid sequence identity. By analyzing the protein primary structures, it has been demonstrated that ALDHs included in the superfamily usually share at least 15% of their sequence [10]. In addition, members of the ALDH superfamily are grouped into families and subfamilies whenever they share around 40% or 60% sequence identity [11,12].

**Figure 1:**
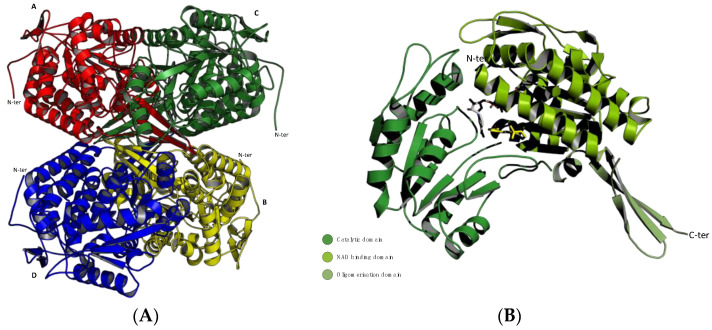
Ribbon representations of the monomeric and oligomeric structure of ALDH1A3. Structures were generated using the PyMOL Molecular Graphics System, Version 1.3 Schrödinger, LLC; PDB code: 5FHZ. (**A**) The ALDH1A3 tetramer with chains (A–D) colored in red, yellow, green, and blue, respectively. The N-terminals (N-ter) are highly mobile and exposed on the surface of the tetramer, while the C-terminals point toward its core. N-t: N-terminus. (**B**) The ALDH1A3 monomer with its three domains indicated by different shadows of green: the NAD binding domain is shown in light green, the catalytic domain in forest green, and the oligomerization domain in olive green. The ligands NAD^+^ and RA are shown as grey and yellow sticks, respectively.

All the structures obtained from crystallization studies showed that ALDH isoenzymes are functional when in dimeric or tetrameric form, depending on which class they belong to (Figure 1A), but globally, they share high structural homology in the monomer unit, which consists of three different domains: the coenzyme binding domain, the catalytic domain, and the oligomerization domain [13] (Figure 1B). The arrangement of these domains gives rise to a funnel-like passage ending in the catalytic site. The upper portion of the funnel-like passage provides the amino acid specificity to the substrate binding, and the highly conserved lower portion binds the cofactor allowing the hydride transfer from the substrate [14].

Overall, these enzymes share a number of highly conserved amino acid residues that take part in both the catalytic processes and the cofactor binding. The most relevant amino acids are Cys302 directly involved in the catalytic activity and Glu268 which acts as a base activating the hydrolytic water molecule. As customary, the reference number of amino acids is from the human ALDH2 sequence [11,14].

The irreversible conversion of aldehydes into carboxylic acids is a highly conserved process in all of the NAD(P)^+^-dependent ALDH enzymes. However, ALDH6A1 has coenzyme A (CoA) as the coenzyme, resulting in a CoA ester as its main product instead of a free carboxyl acid due to the cofactor reduction and its release before the deacylation step [14,15].

**Figure 2:**
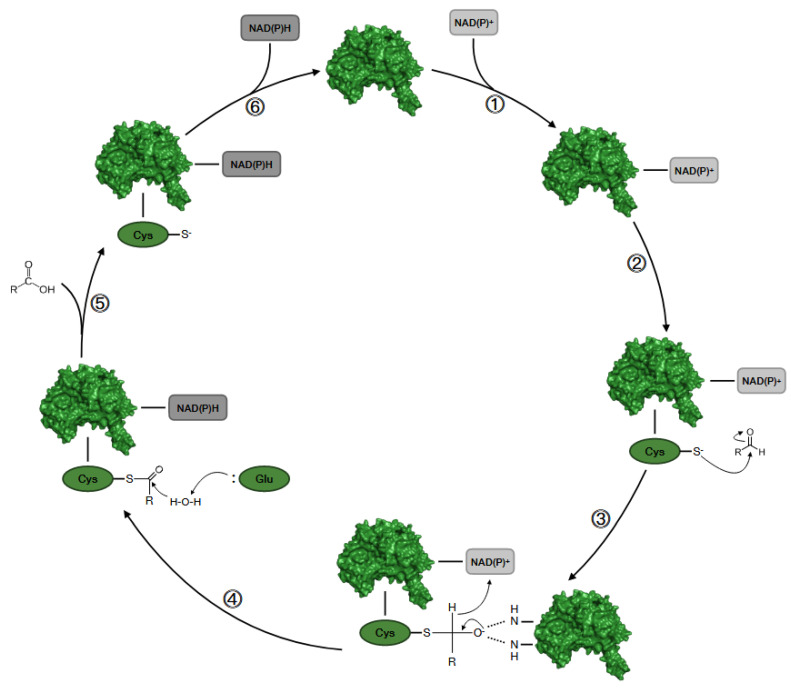
Schematic representation of the catalytic cycle of NAD(P)^+^-dependent ALDH. (1) The enzyme binds the NAD(P)^+^ cofactor; (2) the SH group of the activated Cys-302 attacks the aldehydic group of the substrate; (3) formation of a stabilized oxyanion thiohemiacetal intermediate; (4) formation of a thioacylenzyme intermediate with the formation of NAD(P)H; (5) the substrate is released after the hydrolysis of the thioacylenzyme of carboxyl acid; (6) the enzyme is regenerated upon releasing NAD(P)H and is ready for binding a new molecule of NAD(P)^+^.

The more common NAD(P)^+^-dependent mechanism can be rationalized in six essential steps as shown in Figure 2.

The first step is the binding of NAD(P)^+^ to the corresponding site that strongly stabilizes the complex, induces a conformational change, and activates the nucleophile catalytic residue of Cys302 with a mechanism mediated by Glu268 and a water molecule. Then, the hydride transfer on the cofactor takes place from the thiohemiacetal intermediate, leading to the reduction of the coenzyme to form NAD(P)H. The enzyme is regenerated by the binding of a new molecule of NAD(P)^+^, and the reduced form of the substrate detaches from the binding site [13,16].

## 3. The Human Aldehyde Dehydrogenase 1A Subfamily

The ALDH1A subfamily comprises three different isoenzymes: ALDH1A1, ALDH1A2, and ALDH1A3 [13]. These proteins have the major task of regulating gene expression by controlling the oxidation of *all*-*trans* or 9-*cis* retinal to RA with high specificity. RA, through the binding to two different nuclear hormone receptors, namely retinoid-X receptors (RXRs) and retinoic acid receptors (RARs), plays a crucial role as a tissue differentiation factor for cellular development and morphogenesis. RA’s presence during the developmental processes in embryogenesis regulates neurogenesis, cardiogenesis, and the formation of the eyes and other organs, while also being involved in the cell–cell signaling networks between pluripotent cells [17]. Even though they share over 70% of their primary sequence and mostly recognize a common substrate, ALDH1A isoenzymes maintain separate patterns and substrate preferences, and this highlights the importance of these enzymes in gene expression, as they regulate over 530 genes through RA action [16,18]. In further detail, the isoform ALDH1A3 is a cytosolic homotetramer, and its mRNA can be found also in the nucleus and mitochondria. Each monomer shows a molecular weight close to 56 KDa and is able to catalyze a single oxidation of retinal to RA using NAD^+^ as a cofactor. This isoenzyme, like ALDH1A1 and ALDH1A2, can catalyze both all-*trans*-retinal and 9-*cis*-retinal, but it shows a preferential catalytic activity towards all-*trans*-retinal that is considerably higher compared to ALDH1A1 [16,19]. ALDH1A3 is widely expressed in both embryonic and adult tissues, especially in the fetal nasal mucosa, salivary glands, stomach, kidneys, and breast. It is also involved in the development of several structures of the brain, from eyes and olfactory bulbs to the forebrain and the cerebral cortex [10]. Different studies reported how ALDH1A3 plays a crucial role in glycolysis and gluconeogenesis in oxidizing acetaldehyde as a result of ethanol metabolism through alcohol dehydrogenase (ADH). In addition, ALDH1A3 even intervenes in amino acid metabolism, oxidizing the intermediate aldehydes that form from histidine, phenylalanine, β-alanine, and tyrosine. Finally, several aldehydes derived from lipid peroxidation and cytochrome-mediated metabolism of exogenous substances and drugs are detoxified by ALDH1A3, which contributes to a reduction in oxidative stress and damage. One of the most important tasks of this enzyme is regulating cell apoptosis. Indeed, it was demonstrated that in *ALDH1A3*-knockout mice, mitosis decreases, with RA inducing the expression of pro-apoptotic genes like *caspase*-7 and *caspase*-9 leading to apoptosis [19].

## 4. Structural Comparison between ALDH1A Isoenzymes

In order to understand the activity and function differences between the ALDH1A isoenzymes, a structural comparison could be useful. Analyzing the monomer structure of ALDH1A1, ALDH1A2, and ALDH1A3, a high level of amino acid sequence identity was confirmed. Specifically, the ALDH1A3 monomer shares 71% identity with ALDH1A1 and 72% with ALDH1A2.

**Figure 3:**
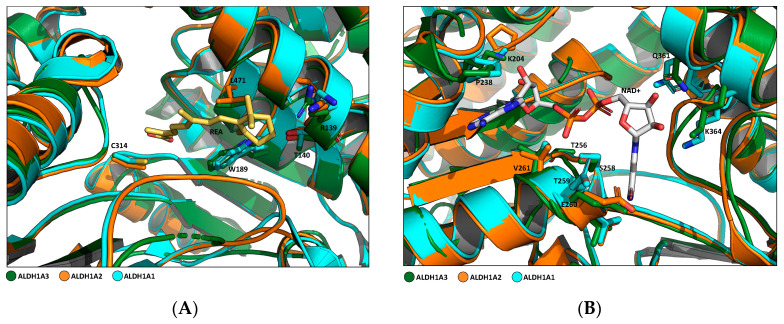
Comparison of RA and NAD binding sites of the three ALDH1As. Structures were generated using the PyMOL Molecular Graphics System, Version 1.3 Schrödinger, LLC; PDB code: 5FHZ. (**A**) Superposition overview of different amino acids involved in RA-ALDH1A3 binding. Side chains of key residues involved in the binding (L471, R139, T140, W189, and C314 (PDB code: 5FHZ)) are shown in forest green sticks. The corresponding residues conserved in ALDH1A2 (PDB code: 6ALJ) and ALDH1A1 (PDB code: 4WB9) are represented by orange and cyan sticks, respectively. RA is shown in yellow sticks. (**B**) Superposition overview of different amino acids involved in NAD^+^-ALDH1A3 binding. Side chains of key residues involved in the binding (K204, P238, T256, S258, T259, E260, V261, Q361, and K364 (PDB code: 5FHZ)) are shown in forest green sticks. The corresponding residues conserved in ALDH1A2 (PDB code: 6ALJ) and ALDH1A1 (PDB code: 4WB9) are represented by orange and cyan sticks, respectively. NAD^+^ is shown in grey sticks.

In addition, several amino acid residues are conserved in the three isoforms, mainly Try189, Gly136, Leu185, Leu471, and Ala473, according to the ALDH1A3 sequence numbering. These amino acids establish van der Waals interactions with the natural ligand RA inside the catalytic site and are conserved in all three proteins. This can explain how RA is their main shared ligand (Figure 3A) [16]. The amino acids involved in the NAD^+^ cofactor binding site are all tightly conserved, both among proteins of the 1A subfamily and in all 19 human ALDH isoforms (Figure 3B). Aside from the proven conserved structure, it has been demonstrated that, especially in the catalytic domain and RA binding site, not all the residues are commonly shared through the isoenzymes. In particular, ALDH1A3 in its binding site located in the substrate access to catalytic pocket presents Phe131, Glu135, and Tyr472, which correspond to Leu 120, Ala 124, and Ser 461 in ALDH1A1 and Tyr137, Gln141, and Asn478 in ALDH1A2, respectively. In addition, in a comparison of ALDH1A3 and ALDH1A1 catalytic tunnel sequences, two significant differences were discovered: Thr315 and Asn469, fundamental in ALDH1A3, are replaced with Ile304 and Gly458 in ALDH1A1. These substitutions have been decisive in obtaining new pharmacological tools, fluorescent probes, and selective inhibitors of ALDH1A3 [4,20,21,22,23].

## 5. ALDH1A3 and Cancers

The proteins encoded by the ALDH1 subfamily play an important role in several human cancers [24]. A list of human tumors where ALDH1A3 expression has been implicated in the generation or growth of the neoplasm is provided in Table 1. Usually, increased ALDH1A3 expression in the tumor compared to that of the corresponding normal tissue indicates enhanced malignity or a worse prognosis (Table 1). However, there are exceptions like in melanoma [25] and non-small-cell lung cancer [26], where ALDH1A3 overexpression is associated with longer survival and a reduced incidence of metastasis.

## 6. ALDH1A3 in Glioma and Glioblastoma

According to the last revision of the Human Protein Atlas [43], all ALDH1A isoenzymes are expressed in the brain with low regional specificity; ALDH1A3 has a higher level of expression in the choroid plexus, but the protein is detectable in all regions of the brain. In the Human Protein Atlas, the levels of the ALDH1A isoenzymes are always not considered prognostic for glioma [43]. However, the same source indicates that lower mRNA levels of ALDH1A1 and ALDH1A3 in high-grade gliomas are associated with an increased proportion of patients surviving more than 3 years [43].

In glioblastoma (GBM), like in many other tumors, ALDH1A3 expression was linked to tumor stem cells [44] and more specifically to cancer stem cells of GBM, with a transcriptomic signature indicated as mesenchymal [31]. Later studies based on multiple samples from the same tumor and single-cell RNA studies have shown that the different transcriptomic subtypes can coexist in the same tumor [45]. Moreover, the number of GBM transcriptomic patterns that show strong indications of representing antagonistic states has been reduced to two: proneural (or neural progenitor-like) and mesenchymal. These states dominate the patterns of phenotypic heterogeneity in GBM [45,46]. Mesenchymal GBM cells are more aggressive and less sensitive to radiotherapy and chemotherapy [47] and are characterized by an increase in ALDH1A3 levels [31,48,49]. Patients affected by GBM richer in cells with a mesenchymal transcriptional phenotype show earlier recurrences and have a shorter survival [20,50]. Tissue samples obtained from relapsing GBM, independent from their initial phenotype, contain a higher amount of cancer cells with a mesenchymal phenotype [51].

In GBM, ALDH1A-positive cells tend to be enriched in the invasive region of the tumor where glioma stem cell niches are more concentrated [50,52]. The presence of ALDH1A3-positive cancer stem cells is also instrumental in maintaining hypervascularization of the stem cell niches by paracrine release of plasminogen activator inhibitor-1 and interleukin-8, which stimulate neovasculogenesis [53].

The treatment of GBM patients with temozolomide (TMZ), the mainstream drug used for the treatment of human GBM, is invariably followed by the selection of cells that are resistant to TMZ. Although the level of ALDH1A3 is lower in TMZ-resistant GBM cells than in cells sensitive to TMZ, the enzyme is still detectable and contributes to the survival of those cells [54,55].

In recurrent GBM tissue that is often largely composed of cells with a mesenchymal phenotype [51], the level of ALDH1A3 is increased [55]. This suggests that ALDH1A3 may be a useful pharmacological target in order to develop therapeutic options for GBM patients whose ominous prognosis is tightly linked with the ability of GBM to relapse [56].

Overexpression of ALDH1A1 has also been described in human high-grade gliomas [57] but is not uniform inside GBM and is typical of cells located at the periphery of the tumors with a classical transcriptional subtype that have the worst prognosis [55].

## 7. ALDH1A3 Functions in Glioma and Glioblastoma Are Multifaceted

In human GBM and other gliomas, ALDH1A3 is involved in different biochemical pathways (Table 2) that may explain the importance of the enzyme for tumor proliferation and survival.

The first and foremost important pathway is related to the involvement of ALDH1A3 in the synthesis of *all*-*trans*-retinoic acid (*at*RA). Two enzymatic reactions are necessary to convert retinol to *at*RA: first, alcohol dehydrogenases (Adhs) (isoenzymes 1, 3, and 4) or retinol dehydrogenases (RDHs 1 and 10) convert retinol to retinaldehyde, and then retinaldehyde may be irreversibly converted by ALDH1A1, ALDH1A2, or ALDH1A3 to *at*RA. ALDH1A1 is less efficient than ALDH1A2 and ALDH1A3 in synthesizing *at*RA [58]. RA in vivo is short-lived (30 min), and enzymes like CYP26B1 contribute to its degradation [62]. Interestingly, the overexpression of CYP26B1, which breaks down *at*RA, has been associated with enhanced survival of African American patients affected by GBM who demonstrate a Karnofsky Performance Score > 80 at diagnosis [63]. On the contrary, enhanced dietary intake of vitamin A [64] and β-carotene [65] is associated with a lower incidence of GBM. Increased production of *at*RA by ALDH1A3 in GBM increases the level of transglutaminase, promoting the survival of cancer cells [49].

Quantitative analysis by high-performance liquid chromatography (HPLC) of the concentration of *at*RA in tissue derived from clinical samples of GBM gave very low or undetectable values in the majority of tumors; in tumors where *at*RA was detectable, the values ranged from 4.4 to 31.2 ng/g tissue [66]. However, these values only reflect the bulk concentration of RA, while local accumulation of retinoids may be far more biologically important but go completely undetected.

The effects of the pharmacological administration or increased local synthesis of *at*RA in GBM have been described both as promoting and inhibiting GBM growth [66,67,68,69]. In humans, the RA signal is transduced by heterodimers of α, β, and γ retinoic acid receptors (RARs) and α, β, and γ retinoid-X receptors (RXRs) whose genes are expressed in GBM [70]. Moreover, RXRs can also form heterodimers with other partners like peroxisome proliferator-activated receptor (PPAR) [71] which is also expressed in GBM [72]. Heterodimers of RAR and RXR act as repressors or activators of gene expression by interacting with corepressors and coactivators after binding the appropriate ligands [73]. RARs bind both *at*RA and 9-*cis*-retinoic acid (9*cis*RA), whereas 9*cis*RA activates only RXR [71]. The presence of RXR “subordination” in heterodimers of RAR and RXR indicates that RXR cannot respond to its ligands unless RAR is already activated [73]. The presence of this complex multilayered network of protein interactions and ligands may explain the different actions ascribed to retinoids in gliomas [66,74].

Another mechanism that is independent of RAR/RXR activation and may explain the importance of ALDH1A3 in gliomas and other tumors has been linked to an increased production of NADH that protects the cell from ferroptosis by the activation of ferroptosis suppressor protein 1 (FSP1). Ferroptosis protection is dependent on NADH generated by ALDH1A3 and is essential for the activation of FSP1 oxidoreductase, which transforms extra-mitochondrial ubiquinone to ubiquinol, counteracting lipid peroxidation and, thus, ferroptosis by reducing peroxyl radicals in phospholipid acyl chains [59,60]. ALDH1A3 in GBM may also directly influence ferroptosis by detoxifying the aldehydes resulting from spontaneous decomposition of lipid peroxides in cell membranes produced by the action of reactive oxygen species [55]. However, ALDH1A3 expression in GBM cells is necessary for the activity of exogenous ferroptosis inductors [54,75].

Another retinol-independent pathway that may be influenced by the enrichment of ALDH1A3 in proliferating cells of GBM and other tumors was initially described in endothelial cells of patients affected by pulmonary arterial hypertension [76]. In those cells, the local ALDH1A3-mediated production of acetate from acetaldehyde in the nucleus increases the acetyl coenzyme A pool available for histone H3 acetylation [76]. In GBM cells, ALDH1A3 turnover is dependent on the ubiquitin–proteasome system. As a result, both incompletely degraded isoforms of ALDH1A3, which partially retain their function, and inactive peptides generated by a more complete proteolysis accumulate in the nucleus [77].

Moreover, the pathogenic variant of histone H3 (H3K27M) that is present in midline diffuse gliomas (MDGs) is responsible for ALDH1A3 overexpression in those tumors [61]. Overexpression of ALDH1A3 in MDG cells is strongly inhibited by the pan-Wnt inhibitor XAV-939. In MDG, ALDH1A3 converts acetaldehyde into acetate, increasing the pool of acetate and CoA in the nucleus that in turn promotes the acetylation of the unmutated Lys27 of histone H3 [61,76].

## 8. Modulation of ALDH1A3 Expression at Transcriptional and Post-Transcriptional Levels

*ALDH1A1* and *ALDH1A3* promoter regulation depends on multiple transcription factors and enhancers [78]. CCAAT enhancer binding protein beta (CEBPβ) binding to a CAAT box in the *ALDH1A3* promoter is responsible for the overexpression of the *ALDH1A3* gene in several cancer cells [34]. In GBM cells, activated NF-kB can bind to the *ALDH1A3* promoter and increase the expression of the gene [79]. The activation of the b-catenin/T cell factor pathway also increases the transcription of ALDH1A3 [76]. The *ALDH1A3* promoter is often hypermethylated compared to normal tissue in cell lines derived from lung [80], breast [80], colon [80], prostate [80], and cervical [28] cancers. Hypermethylation of the *ALDH1A3* promoter in GBM patients is associated with a low expression of ALDH1A3 and a better prognosis [81]; the same seems to be true for non-muscle-invasive bladder cancer [42].

On the contrary, certain carcinogens present in the environment like benzopyrene increase the methylation of Lys36 of histone H3 (H3K36me3) associated with the *ALDH1A3* gene, which stimulates *ALDH1A3* expression [82]. In colorectal cancer, the *ALDH1A3* promoter is overactivated by *trans*-3-indole acrylic acid (*t*3IDA). *t*3IDA is a tryptophan metabolite produced by *Peptostreptococcus anaerobius* that may be present in the human intestinal microbiota. *t*3IDA binds and activates the aryl hydrocarbon receptor that binds to the ALDH1A3 promoter and upregulates its transcription [59]. An increase in ALDH1A3 is necessary for colorectal carcinogenesis induced by *t*3IDA [59,83] In this pathway, one of the major roles of ALDH1A3 is NADH production, which is necessary for FSP1’s inhibitory activity on ferroptosis [59,83]. The aryl hydrocarbon receptor is present in GBM and malignant gliomas, but it is still not known if *t*3IDA also plays a role in its activation in these tumors with an effect on ALDH1A3 transcription as described in colorectal cancer.

Another level of control on ALDH1A3 expression in gliomas and other cancers comes from miRNA targeting *ALDH1A3* transcripts: miR-187 and miR-1301-3p downregulate *ALDH1A3* expression in prostate cancer [84,85], miR-200-ZEB1/SANI2 inhibits *ALDH1A3* expression in colorectal cancer cells [86], miR-548 downregulates *ALDH1A3* expression in hepatocellular carcinoma and macrophages [32], and miR320b and miR-4524b-5p do the same in human high-grade gliomas and GBM, resulting in decreased proliferation and increased resistance of glioma cells to irradiation [44,87].

Post-transcriptional modifications can also modulate the stability and transcriptional efficiency of *ALDH1A3* mRNAs. Methyltransferase-like 3 (METTL3) is an RNA methyltransferase capable of methylating adenosine contained in mRNA in position 6 and generating N^6^-methyladenosine, the most extensive endogenous modification of mRNA. In head and neck squamous cell carcinoma, *ALDH1A3* mRNA is one of the mRNAs that is most extensively modified by METTL3 [88]. METTL3 also promotes the proliferation and self-renewal of human GBM stem cells [89].

The intracellular level of ALDH1A3 is also controlled by protein degradation triggered by polyubiquitylation. In GBM cells with mesenchymal differentiation, the level of polyubiquitylation of ALDH1A3 is kept low by ubiquitin-specific protease 9X (USP9X), which acts as an efficient and specific deubiquitinase of ALDH1A3 [90]. USP9X depolyubiquitylation of ALDH1A3 effectively increases the half-life of ALDH1A3 in vitro and in vivo, helping the tumor maintain high levels of tumorigenic stem cells [90].

## 9. ALDH1A3 in Pleural Mesothelioma

Pleural mesothelioma (PM) is classified by the European Union as a rare disease. PM is an aggressive cancer that develops in the protective lining around the lungs, and it is caused by asbestos exposure. While there is an overall decreasing incidence trend in highly developed countries, an increasing trend was observed in other countries due to continuing asbestos use and environmental pollution [91]. PM is an orphan disease in terms of understanding pathogenic mechanisms and effective therapeutic approaches. The high number of PM patients refractory to first-line chemotherapy based on cisplatin and pemetrexed, as well as the increasing rates of second-line therapy failure, suggest the urgent need for novel mechanism-based therapies. In the absence of strategic oncogenic drivers for stratifying PM patients, it is crucial to identify signaling pathways that drive PM development and sustain its growth [92,93]. Recently, two immune-checkpoint blockers (ICBs), nivolumab and ipilimumab, have been approved as a frontline treatment option for unresectable PMs; despite promising clinical results, the mechanisms of primary and acquired resistance remain to be better elucidated [94,95].

An analysis of the cancer genome atlas (TCGA) database involving 84 PM patients revealed that high *ALDH1A3* expression is significantly associated with poorer prognosis [96]. This finding is confirmed by data demonstrating that in vitro treatment of stable and primary PM cell lines with cisplatin and pemetrexed triggered the emergence of drug-resistant PM cell subpopulations that exhibited high expression of mesenchymal markers and *ALDH1A3*, together with a high ALDH activity (ALDH^bright^). The results obtained from Affymetrix gene expression profiling analyses of ALDH^bright^ and ALDH^low^ PM subpopulations revealed that 924 genes are differentially regulated according to ALDH activity status.

Signaling pathway analysis indicated that among the most upregulated pathways in the ALDH^bright^ subpopulation, there is an enrichment of the NF-κB-related pathway. This is in accordance with the crucial role exerted by NF-κB in the survival and growth of chemoresistant cells [96]. Canino and colleagues described that in ALDH^bright^ PM drug-resistant subpopulations, the complex signal transducer and activator of transcription 3 (STAT3)-NF-κB is involved in the regulation of *ALDH1A3* mRNA transcription. They demonstrated that pSTAT3(Tyr705)-NF-κB(p65) complex is required for the repression of DNA Damage Inducible Transcript 3 (*DDIT3*); this led to an instability of the complex DDIT3-CEBPβ and an increased occupancy of *ALDH1A3* promoter by CEBPβ, resulting in high levels of *ALDH1A3* expression. Disruption of STAT3-NF-κB complex stability with the compound butein significantly reduced *ALDH1A3* expression, enhanced DNA damage response, and improved chemosensitivity in vitro and in vivo [34,96].

**Figure 4:**
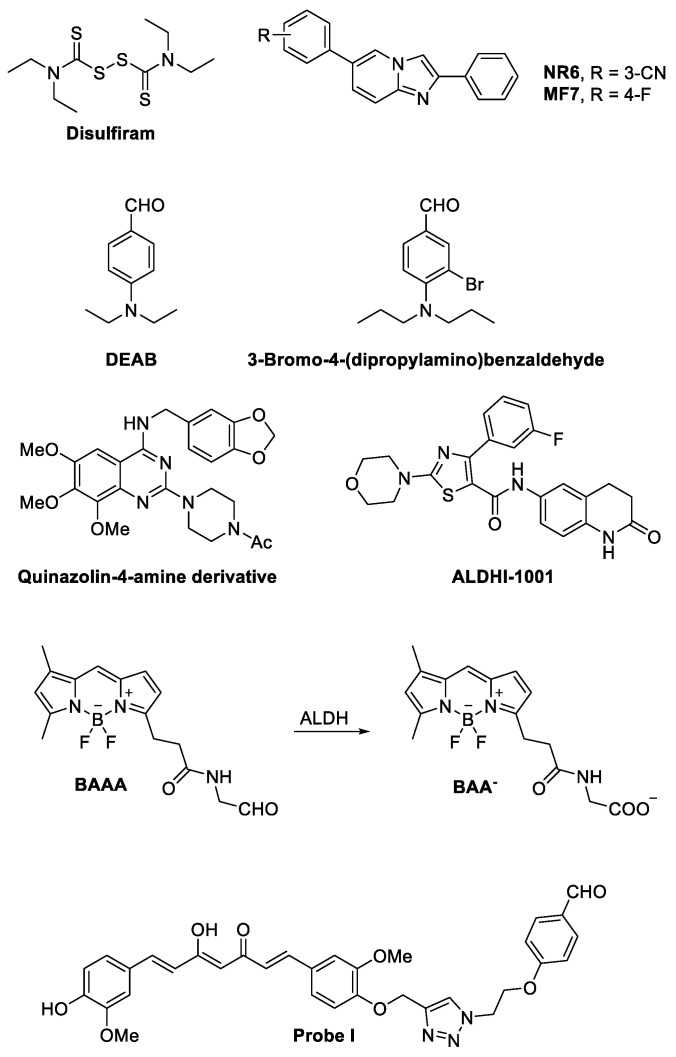
Structures of ALDH inhibitors and an ALDH-inhibiting fluorescent probe. For 4,4-difluoro-4-bora-3a,4a-diaza-*s*-indacene (BODYPY)-aminoacetaldehyde (BAAA), the corresponding benzoic acid generated by ALDH enzymatic activity on BAAA is also reported.

Moreover, Boumya and colleagues reported that NR6 (Figure 4), a highly potent and selective ALDH1A3 inhibitor, hindered the growth of ALDH1A3-positive PM cells cultured in 3D as multicellular spheroids (MCSs). NR6 induced a senescent growth arrest and led to the expression of cyclin-dependent kinase inhibitor 2A (CDKN2A). They demonstrated that NR6 caused the intracellular accumulation of the toxic aldehyde malondialdehyde (MDA), resulting in DNA damage and a global reduction in total NAD in PM MCSs. Furthermore, they reported that NR6 treatment stimulated *IL6* expression but significantly inhibited the expression and release of IL-8, thereby impairing neutrophil recruitment [35].

## 10. ALDH1A3-Selective Inhibitors

Inhibitors of ALDH1A3 may be valuable in developing new therapies for patients with cancer, obesity, diabetes, and cardiovascular disorders. Moreover, ALDH1A3 inhibitors may also modulate the immune response and enhance T-cell infiltration of tumors, resulting in the inhibition of tumor growth [97].

Current studies on the effects of ALDH1A inhibitors on the growth and proliferation of cancer cells have been carried out using compounds that had little isoform specificity and often were simultaneously interfering with other members of different classes of the human ALDH superfamily. A common example is disulfiram (Figure 4), a clinically approved inhibitor of ALDH having activity on multiple ALDHs (Table 3) [98], which was employed in combination with copper in a multicenter phase II study on recurrent TMZ-resistant GBM without positive results [99]. Similarly, no significant clinical results were obtained by the addition of disulfiram to cisplatin in the therapy of refractory germ cell tumors [100]. On the contrary, the addition of disulfiram to chemotherapy for the treatment of metastatic non-small-cell lung cancer appeared to prolong patient survival [101]. The contradictory results obtained in trials where disulfiram was added to standard chemotherapy to treat cancer may be in part due to the prodrug nature of the molecule, its low specificity, and its rapid metabolism in vivo [102]. These considerations do not necessarily indicate that inhibiting ALDH in tumors is not worth pursuing.

On the contrary, many research groups have recently invested much effort in the development of ALDH1A inhibitors with high specificity for the different isoforms, which are currently being tested in preclinical models and, hopefully, could reach the stage of a clinical trial in the foreseeable future. In this context, new selective ALDH1A3 inhibitors have been recently developed starting from structurally different compounds [20,23,86,103,104,105]. Among them, NR6 (Figure 4, Table 3) emerged with a potent and selective inhibitory profile (IC_50_ = 5.3 ± 1.5 µM on the recombinant enzyme). Interestingly, NR6 is able to bind to a specific ALDH1A3 residue (Tyr472) in the catalytic tunnel not conserved in the other subfamily members, as demonstrated by X-ray crystallographic analysis supported by molecular docking simulations. Moreover, NR6 demonstrated in vitro cytotoxic activity against GBM and colorectal cancer cells in the submicromolar range [23]. Further structural modification of N6 led La Motta and coworkers to identify MF7 (Figure 4), a fluoro derivative showing a micromolar IC_50_ (=22.8 ± 1.6 μM, Table 3) on recombinant human ALDH1A3 in enzymatic assays and antiproliferative activity in the breast cancer MDA-468 cell line in vitro [106].

More recently, ALDH1A3 inhibitors with a substituted benzaldehyde scaffold have been developed by chemical modification of 4-(diethylamino)benzaldehyde (DEAB, Figure 4) (micromolar IC_50_ on the recombinant enzyme, Table 3), a known ALDH substrate employed in the Aldefluor^TM^ assay, with some compounds showing antiproliferative activity in vitro against prostate cancer tumor cells when added to the culture medium in the micromolar range. Among them, although not selective for 1A3 isoform, 3-bromo-4-(dipropylamino)benzaldehyde (Figure 4) (IC_50_ = 0.63 ± 0.02 µM on the recombinant enzyme, Table 3) showed a potent antiproliferative activity [105]. A selective ALDH1A3 inhibitor, a quinazolin-4-amine derivative (Figure 4, Table 3), was recently reported by Kamiyama et al. to possess potent inhibitory activity in both enzymatic (IC_50_ = 0.0640 µM on the recombinant enzyme) and Aldefluor^TM^ assays in breast cancer MDA-MB231 cells [103,104]. Also, different 6-substituted-3,4-dihydroquinolin-2(1*H*)-ones, whose representative ALDHI-1001 is depicted in Figure 4, were reported to inhibit selectively ALDH1A3 (Table 3) [107].

None of the new specific ALDH1A3 inhibitors have been extensively tested in preclinical models. However, these studies are underway, and hopefully, they will lead to the identification of potentially useful compounds for further testing in clinics.

**Table 3:** Reported inhibitors of ALDH1A3 and their enzymatic inhibition and selectivity data.

Compound	Enzymatic Inhibition (hALDH1A3)	Selectivity (Enzymatic Assay)	Ref.
Disulfiram	n.a.	IC_50_ = 0.13 ± 0.10 µM on ALDH1A1; IC_50_ = 3.40 ± 0.71 µM on ALDH2	[98]
NR6	IC_50_ = 5.3 ± 1.5 µM; K_I_ = 3.7 ± 0.4 µM	InhIb.% = 11.3 at 25 µM on ALDH1A1; not active at 25 µM on ALDH1A2	[22,23]
MF7	IC_50_ = 22.8 ± 1.6 µM	not active at 25 µM on ALDH1A1 and ALDH1A2	[22]
DEAB	IC_50_ = 10.4 ± 1.0 µM. IC_50_ = 4.27 µM	IC_50_ = 0.48 ± 0.06 µM on ALDH1A1; IC_50_ = 5.67 ± 0.66 µM on ALDH3A1. IC_50_ = 23.9 µM on ALDH1A1; IC_50_ > 100 µM on ALDH1A2; IC_50_ = 2.73 µM on ALDH3A1.	[103,105]
3-Bromo-4-(dipropylamino)benzaldehyde	IC_50_ = 0.63 ± 0.02 µM	IC_50_ = 7.08 ± 0.70 µM on ALDH1A1; IC_50_ = 8.00 ± 1.56 µM on ALDH3A1.	[105]
Quinazolin-4-amine derivative	IC_50_ = 0.0640 µM	IC_50_ = 45.3 µM on ALDH1A1; IC_50_ > 100 µM on ALDH1A2 and ALDH3A1	[103]
3,4-Dihydroquinolin-2(1H)-one representative (ALDHI-1001)	IC_50_ < 0.1 μM	IC_50_ ≥ 100 μM on ALDH1A1, ALDH1A2, and ALDH2	WO 2022/123039 A1 (patent) [107]
Probe I	*K_I_* = 0.880 μM	n.a.	[4]

## 11. ALDH1A3 Ligands as Tumor Markers In Vivo

The long-term interest in developing new ALDH substrates was not only motivated by their potential pharmacological usefulness as inhibitors or activators of ALDH enzymatic activity but also by their extensive use in labeling and selecting specific cell populations.

The first fluorescent non-toxic substrate for ALDHs was 4,4-difluoro-4-bora-3a,4a-diaza-*s*-indacene (BODYPY)-aminoacetaldehyde (BAAA, Figure 4) [108]. BAAA is a compound that passively diffuses into intact cells and is trapped inside the cells after transformation by ALDHs into the corresponding negatively charged benzoic acid (BAA^−^, Figure 4) [108]. It was originally developed for isolating primitive human hematopoietic cells, but after becoming commercially available under the name Aldefluor^TM^, it was used for labeling and isolating cancer stem cells [109]. However, although Aldefluor^TM^ is still widely used for stem cell isolation, it is not specific for a single ALDH enzyme or a single ALDH subfamily but may be metabolized by at least 9 (ALDH1A1, ALDH1A2, ALDH1A3, ALDH1B1, ALDH2, ALDH3A1, ALDH3A2, ALDH3B1, and ALDH5A1) of the 19 different human ALDH isoforms [110]. Moreover, Aldefluor^TM^ interactions with ABC transporters induce high efflux rates of the molecule, reducing the concentration of the tracer inside the cells to a level that may produce false negative results even if the cells are expressing one or more ALDH1A isoforms [111].

These and other issues that affect other fluorescently labeled aminoacetaldehyde derivatives currently available as labels for ALDH1A-expressing cells hamper their use as specific “tools” for the detection and accurate removal of high-grade gliomas and other tumors during surgery. Fluorescence-guided resection of malignant gliomas is a rapidly evolving technique that is currently based on the injection of fluorescent markers like fluorescein and 5-aminolevulinic acid that accumulate in the tumor by unspecific mechanisms [112,113,114]. These same untargeted mechanisms that lead to glioma in vivo labeling by fluorescein can be affected by unknown factors potentially leading to both false negative and false positive errors in tumor tissue identification during surgery.

Capitalizing on our previous experience in developing selective inhibitors of ALDH1A3 [21,22,23], we have developed two different selective ALDH1A3 fluorescent probes: Probe I (Figure 4) and II, with a curcumin scaffold-based nature. P I, besides inhibiting the ADLH1A3 in the nanomolar range (*K_I_* = 0.880 μM on recombinant human enzyme), may fluorescently label experimental high-grade gliomas growing in vivo orthotopically inside the hemispheres of mice brain [4]. In the same experiments, we also found that isolated tumor cells adjacent to the main tumor mass were labeled [4]. This last observation suggests that contrary to fluorescein diacetate, the most common fluorescent probe used for labeling GBM during neurosurgery, the ALDH1A3 substrates can also label tumor cells contained in the brain adjacent to the tumor area. Those cells are responsible for GBM relapse [115], and their identification potentially allows for a more complete removal of the tissue invaded by glioblastoma.

## 12. Conclusions

Increased expression of ALDH1A3 in GBM and many other cancers is now confirmed by multiple approaches by different authors. While the role of ALDH1A3 in the synthesis of *t*RA from retinaldehyde is undoubted, the RA-controlled pathways are certainly not the only ones explaining ALDH1A3 overexpression in GBM and cancer. Both local acetate production from acetaldehyde and NAD(P)H generation by ALDH1A3 activation also seem to play an important role in the growth and survival of gliomas and other cancers.

If we consider the versatility of ALDH1A isoforms in processing different substrates, it is important to investigate if other substrates, apart from retinaldehyde and acetaldehyde, contribute in vivo to the pro-carcinogenic activity of ALDH1A3.

Moreover, considering the new opportunities that the new ALDH1A3 inhibitors and fluorescent probes offer, it is now important to test them in appropriate experimental models that will allow the measurement of their activity and specificity in vivo. All of this will increase our confidence in ALDH1A3 as a marker and a key enzyme targeting some of the most aggressive and still hopeless cancers like GBM and mesothelioma.

## Figures and Tables

**Table 1:** Level of ALDH1A3 in human tumors and its role as prognostic factor in cancer.

Cancer	Level of ALDH1A3	Role in Cancer	Source	References
Breast cancer	High	Negative prognostic value	Human/mouse	[27]
Cervical cancer	High	Negative prognostic value	Human	[28]
Colorectal cancer	High	Negative prognostic value	Human/mouse	[29]
Cholangiocarcinoma	High	Negative prognostic value	Human	[30]
Glioblastoma	High	Negative prognostic value	Human/mouse	[31]
Hepatocellular carcinoma	High	Negative prognostic value	Human	[32]
High-grade serous ovarian cancer	High	Negative prognostic value	Human	[33]
Melanoma	High	Positive prognostic value	Human	[25]
Mesothelioma	High	Negative prognostic value	Human	[34,35]
Neuroendocrine tumor	High	Malignancy indicator	Human	[36]
Pancreatic PNET	High	Negative prognostic value	Human	[37]
Non-small-cell lung cancer	High	Positive prognostic value	Human	[26]
Prostate cancer	High	Enhanced metastasis	Human	[38,39]
Renal cancer	High	Progression	Human	[40]
Thyroid cancer (papillary)	High	Progression	Human	[41]
Bladder cancer	High	Positive prognostic value	Human	[42]

**Table 2:** ALDH1A3-dependent biochemical pathways in GBM.

#	ALDH1A3-Dependent Pathways	Ref.
I	Synthesis of *all*-*trans*-retinoic acid	[58]
II	NAD(P)H production	[59,60]
III	Detoxification of aldehydes derived from lipid peroxides	[55]
IV	Acetate production favors histone H3 acetylation	[20,61]

## Data Availability

All data are available within the paper and the cited references.
